# CHD4 and NOX4 expression in thyroid tumor tissues

**DOI:** 10.37349/etat.2026.1002363

**Published:** 2026-03-25

**Authors:** Salma Fenniche, Mohamed Oukabli, Yassire Oubaddou, Allaoui Mohamed, Mohamed Reda Elochi, Abir Alghuzlan, Abdelilah Laraqui, Nadia Dakka, Youssef Bakri, Corinne Dupuy, Rabii Ameziane El Hassani

**Affiliations:** IRCCS Istituto Romagnolo per lo Studio dei Tumori (IRST) “Dino Amadori”, Italy; ^1^Laboratory of Biology of Human Pathologies (BioPatH), Faculty of Sciences, Mohammed V University in Rabat, Rabat 1014, Morocco; ^2^Gustave Roussy Cancer Campus, Pavillon de Recherche N_2, 94805 Villejuif, France; ^3^Faculty of Medicine and Pharmacy, University Paris-Saclay, 91400 Orsay, France; ^4^Unité Mixte de Recherche UMR9019 Centre National de la Recherche Scientifique, Pavillon de Recherche N_2, 94805 Villejuif, France; ^5^Service of Anatomical Pathology, Military Hospital of Instruction Mohammed V (HMIMV-R), Rabat 1014, Morocco; ^6^Faculty of Medicine and Pharmacy, Mohammed V University in Rabat, Rabat 10001, Morocco; ^7^Sequencing Unit, Laboratory of Virology, Center of Virology, Infectious and Tropical Diseases, Royal School of Military Health Service, Mohammed V Military Teaching Hospital, Rabat 10001, Morocco

**Keywords:** chromodomain-helicase-DNA-binding protein 4 (CHD4), papillary thyroid carcinomas, NADPH oxidase 4, *BRAF^V600E^* mutation, molecular marker, epigenetic

## Abstract

**Aim:**

Chromodomain-helicase-DNA-binding protein 4 (CHD4) is a core NURD remodeling complex ATPase that plays a crucial role as a gene repressor. Its overexpression has been reported in several cancers. In papillary thyroid carcinomas (PTCs), CHD4 is overexpressed and associated with aggressive features of the tumor, such as proliferation, migration, and epithelial-mesenchymal transition (EMT). We previously showed in PTCs that NADPH oxidase NOX4 expression is positively regulated by *BRAF^V600E^* mutation, which is the most aggressive alteration in PTCs. In this retrospective study, we wondered whether there is a link between CHD4 and NOX4 protein expression in malignant thyroid tissues.

**Methods:**

We explored CHD4 protein expression by immunostaining analysis in 86 human thyroid tissues: 44 thyroid tumor tissues [28 classical forms of PTCs (C-PTCs), 13 follicular variants of PTCs (F-PTCs), and three anaplastic thyroid carcinomas (ATCs)] and 42 of their normal adjacent tissues (NATs). The detection of *BRAF^V600E^* mutation was performed using Sanger sequencing and digital droplet PCR. Statistical analyses were conducted using GraphPad Prism 8 software. Various tests were used to assess the statistical relevance of different correlations, such as the chi-square test, Fisher’s exact test, and the Pearson correlation coefficient. A *p*-value of less than 0.05 indicates statistical significance.

**Results:**

The CHD4 protein expression analysis with already published data from our group (*BRAF^V600E^* status and NOX4 expression) reveals a highly significant level of CHD4 protein expression in C-PTCs compared to F-PTCs and ATC. Importantly, 70% of C-PTCs-*BRAF^V600E^* overexpress CHD4 at the protein level, confirming the positive correlation between the CHD4 expression and *BRAF^V600E^* mutation. Furthermore, a high level of CHD4 is associated with the presence of capsular breach and vascular emboli, affirming the involvement of CHD4 in thyroid tumor aggressiveness. Interestingly, we showed for the first time, to our knowledge, a positive correlation between CHD4 and NOX4 protein expression in malignant thyroid tissues.

**Conclusions:**

The results of this study suggest that CHD4 could be used as a complementary molecular marker to improve the diagnosis and the management of PTCs-*BRAF^V600E^*.

## Introduction

Papillary thyroid carcinomas (PTCs) represent approximatively 80% of thyroid cancers. PTCs are characterized by several genetic alterations, the most frequent being the *BRAF^V600E^* mutation [1, 2]. This mutation is known to be associated with thyroid tumor aggressiveness [1, 3–5]. The molecular mechanism by which the *BRAF^V600E^* mutation induces genetic instability is associated with constitutive activation of the MAPK signaling pathway [1, 3–5]. Furthermore, epigenetic deregulation has been observed in PTCs driven by *BRAF^V600E^* mutation, in particular, the hypermethylation of the promoters of certain iodide-metabolizing genes such as natrium iodide symporter “NIS’’ which is related to resistance to radioactive iodine therapy [6–9]. We recently showed by immunohistochemical analysis that NADPH oxidase 4 (NOX4), a constitutive generator of reactive oxygen species (ROS), is highly expressed in the classical form of PTCs (C-PTCs) (92.9% with a high level of NOX4 protein) compared to their normal adjacent tissues (NATs) (100% with a low level of NOX4 protein) [10]. Additionally, this overexpression of NOX4 was positively correlated with the *BRAF^V600E^* mutation (100% of PTCs-*BRAF^V600E^* present a high level of NOX4 protein) [10]. Likewise, at the transcriptional level, Azouzi et al. [8] have previously shown in thyroid tumor cell lines driven by *BRAF^V600E^* that NOX4 is positively regulated by the *BRAF^V600E^* oncogene and that NOX4-derived ROS contribute to the repression of NIS. NOX4 depletion reverses this effect, suggesting the involvement of an epigenetic mechanism. ROS are well known to modulate chromatin structure and epigenetic regulators, thereby influencing gene expression programs involved in tumor progression.

CHD4 (Chromodomain-helicase-DNA-binding protein 4), a subunit of the NuRD (nucleosome remodeling and deacetylation) complex, is an epigenetic regulator of gene expression [11]. CHD4 dysregulation is associated with various types of cancers, including glioblastoma [12], breast cancer [13–15], colorectal [16], lung [17], rectal cancer [18], and PTCs [19]. It is involved in several aspects of carcinogenesis, such as proliferation, cell cycle regulation, migration, epithelial-mesenchymal transition (EMT), and DNA damage repair [16, 19]. Pratheeshkumar and his colleagues [19] showed an overexpression of CHD4 in PTCs. This overexpression was found to be associated with tumor aggressiveness. Indeed, in BCPAP and TPC-1 thyroid tumor cell lines, CHD4 negatively regulates the expression of genes involved in migration and EMT, including E-cadherin [19]. Interestingly, it has been shown that CHD4 is recruited to DNA damage sites in response to oxidative damage and double-strand breaks [20, 21]. Therefore, CHD4 recruitment to sites of ROS-induced DNA damage suggests that increased oxidative stress may enhance CHD4 engagement and activity. Therefore, *BRAF^V600E^*-induced NOX4 overexpression and ROS production could indirectly favor CHD4 activation or stabilization at the chromatin level. In this retrospective study, we investigate the CHD4 expression at the protein level in 86 human thyroid tissues (44 malignant tissues and 42 NATs), and we perform a comparative analysis of its expression with our recently published data regarding *BRAF^V600E^* status and NOX4 protein expression [10]. Our results showed that CHD4 protein expression was significantly increased in C-PTCs compared to their NATs, with 50% (14/28) of C-PTCs showing a high expression of CHD4. Additionally, our results highlight the association between the high levels of CHD4 and aggressive features of thyroid cancer, notably the presence of vascular emboli (57.1%) and capsular breach (54.5%). Interestingly, we establish for the first time, to our knowledge, a positive correlation between CHD4 and NOX4 protein expression in thyroid carcinomas, suggesting a potential cooperative role of both NOX4 and CHD4 in epigenetic instability associated with thyroid cancer.

## Materials and methods

### Retrospective study

This research was approved by the Ethics Committee for Biomedical Research (CERB) of the Faculty of Medicine and Pharmacy in Rabat under approval number 52/20.

In this retrospective analysis, we collected 86 samples from thyroid cancer patients diagnosed from January 2015 to December 2021 at the Department of Anatomical Pathology (Military Hospital of Instruction Mohammed V (HMIMV), Rabat, Morocco). Our cohort comprised 44 malignant thyroid tumor samples containing 41 PTCs (28 C-PTCs, 13 F-PTCs), 3 anaplastic thyroid carcinomas (ATCs), and 42 of their NATs. Histologically, thyroid tissues were classified by an experienced pathologist based on the classification of the World Health Organization (WHO) [22]. Additionally, the validation of retrospective diagnosis was performed by experienced pathologists from both Gustave Roussy Cancer Institute (France) and HMIMV in Rabat (Morocco). The FFPE block was selected as previously described [10].

### Immunohistochemical staining

Immunohistochemical staining was performed according to the manufacturer’s ‘Dako EnVision™ FLEX Kit (K8000)’ protocol using the Autostainer Link 48 device (Agilent). Concerning NOX4, we will explore our results, which have already been published and obtained using rabbit polyclonal anti-NOX4 (ab154244; Abcam) [10]. CHD4 immunostaining was performed using a mouse monoclonal anti-CHD4 (clone 3F2/4, MABE-455; Sigma-Aldrich), whose immunogen is a linear peptide corresponding to human CHD4. CHD4 staining was validated on malignant thyroid tissue (FFPE block) after testing diverse Ab dilutions in EnVision™ FLEX Antibody Diluent (1:50, 1:100, 1:250). Counterstaining of immunohistochemistry (IHC)-stained tissue was performed using EnVision™ FLEX Hematoxylin. Generally, trained pathologists use a score of staining intensity, which might be negative (0), weak (1), moderate (2), and strong (3). In this study, we calculated the hit score (H-score) for both NOX4 and CHD4 by multiplying the percentage of stained cells by the score of staining intensity for each sample. The H-score values range from 0 to 300. The H-score cut-off of ≥ 60 was determined using the median H-score value of the study cohort, a commonly adopted and statistically sound approach to dichotomize continuous immunohistochemical data. In our study, the H-score range was limited to 200. Therefore, we retained the median-based cut-off, as it minimizes classification bias and ensures balanced comparisons between subgroups. Additionally, the pathologists determined the subcellular localization of CHD4 protein in malignant thyroid tissues and their adjacent normal tissues presented on the same IHC slide for each sample. Finally, slides were scanned as previously described [10].

### Mutational analysis

In this study, we explore our previously published results [10]. As a reminder, we used Sanger sequencing and digital droplet PCR (ddPCR) for the detection of the *BRAF^V600E^* mutation after genomic DNA extraction. Genomic DNA was extracted from FFPE tissue sections (8 sections of 10 µm each) using ‘QIAamp DNA FFPE Tissue Kit (Qiagen)’ according to the manufacturer’s instructions. The concentrations and quality of extracted genomic DNA were obtained using the Implen NanoPhotometer N60. Genomic DNA isolated from malignant thyroid tissue was subjected to conventional PCR using the HotStart Taq polymerase (Qiagen). DNA was amplified using the following primers specific for BRAF Exon 15: sense 5'-TCA TAA TGC TTG CTC TGA TAG GA-3' and antisense 5'-GGC CAA AAA TTT AAT CAG TGG A-3'. The PCR products were analyzed on 1.5% agarose gel electrophoresis and visualized under UV-light. The positive samples for *BRAF^V600E^* were bidirectionally sequenced with Sanger direct sequencing using the BigDye™ Terminator cycle sequencing kit (Applied Biosystems) after purification with Exo-SAP at the UATRS Platform (CNRST, Rabat, Morocco). DNA sequences obtained were analyzed using the SeqScape^®^ software v2.7 (Applied Biosystems, Waltham, MA, USA). Samples were also sequenced using ddPCR after DNA genomic extraction with the Maxwell^®^ RSC DNA FFPE Kit, according to the manufacturer’s instructions (ASB1450) at the platform of molecular medicine (Gustave Roussy Cancer Institute, France). The copy number of *BRAFwt* and *BRAF^V600E^* mutation in each sample was noted.

### Statistical analysis

Statistical analyses were executed by GraphPad Prism 8 software. Different tests were used to assess the statistical relevance of several correlations, including Fisher’s exact test, chi-square test, and Pearson correlation coefficient. A *p*-value of < 0.05 indicates statistical significance.

## Results

### Clinicopathological characteristics of patients

Our study population confirmed a female predominance (84.1%) (Table 1). The median age of patients was 42.5 years at the time of diagnosis (with a standard error of 2.4 years). Histologically, 63.6% (28/44) of cases were C-PTCs, while 29.6% (13/44) were F-PTCs, and 6.8% (3/44) were ATC (Table 1). In terms of aggressiveness, about 61.3% (27/44) of tumors present a size larger than 1 cm, 15.9% (7/44) had vascular emboli, 25% (11/44) had a capsular breach, and 6.8% (3/44) present node lymph metastasis (Table 1).

**Table 1:** Clinicopathological characteristics of the cohort population (*n* = 44).

**Clinico-pathological parameters**		***n* (%)**
**Gender**	Female	37/44 (84.1%)
	Male	7/44 (15.9%)
**Age**	Median ± standard error	42.5 ± 2.4
**Histological variant**	C-PTCs	28/44 (63.6%)
	F-PTCs	13/44 (29.6%)
	ATCs	3/44 (6.8%)
**Tumor size**	≤ 1 cm	6/44 (13.7%)
	> 1 cm	27/44 (61.3%)
	Missing data	11/44 (25%)
**Vascular emboli**	Presence	7/44 (15.9%)
	Absence	37/44 (84.1%)
**Capsular breach**	Presence	11/44 (25%)
	Absence	33/44 (75%)
**Lymph node metastasis**	Presence	3/44 (6.8%)
	Absence	30/44 (68.2%)
	Missing data	11/44 (25%)

C-PTCs: classical form of papillary thyroid carcinomas; F-PTCs: follicular variant of papillary thyroid carcinomas; ATCs: anaplastic thyroid carcinomas.

### CHD4 protein expression in thyroid tumor tissues

**Figure 1:**
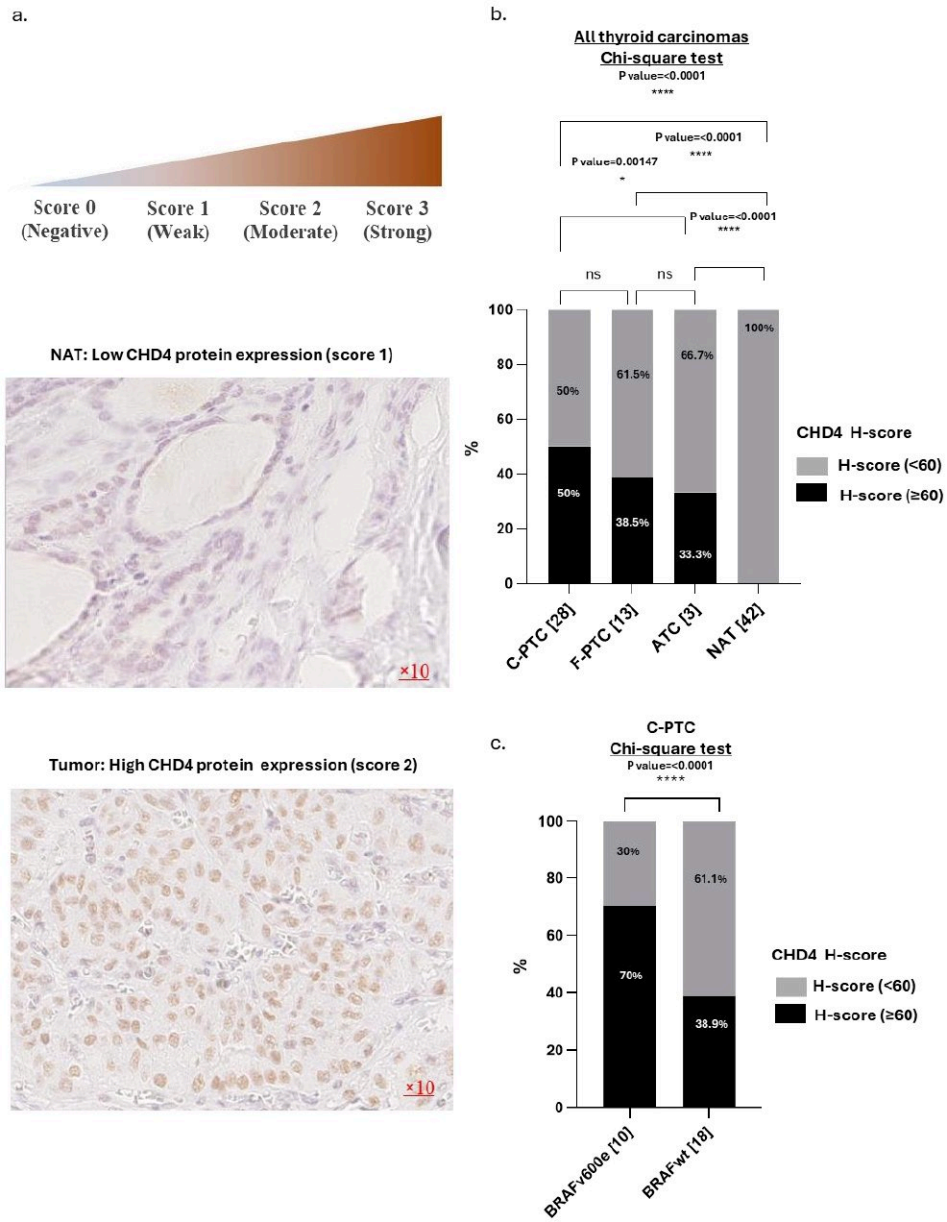
**CHD4 protein expression in thyroid carcinomas. a.** Representative example of PTC with high expression of CHD4 protein and its NAT with low expression of CHD4 protein (Magnification ×10). **b.** Comparative analysis of CHD4 protein expression in human thyroid carcinomas [*n* = 44 (28 C-PTCs, 13 F-PTCs, 3 ATCs)] and their NATs (*n* = 42). Percentage calculated according to the number of each histological variant. The H-score was calculated by multiplying the score of staining by the percentage of stained cells. Two H-scores are attributed to the level of CHD4 protein expression. Score < 60 and ≥ 60. The H-score < 60 represents an absence or low expression of CHD4 protein. The H-score ≥ 60 represents a mean or/and high expression of CHD4 protein. **c.** Association between BRAF profile and CHD4 expression in C-PTCs [*n* = 28 (10 *BRAF^V600E^* and 18 *BRAFwt*)]. The percentage is calculated according to the number of each BRAF profile. The statistical significance is confirmed by a *p*-value < 0.05. CHD4: chromodomain-helicase-DNA-binding protein 4; PTCs: papillary thyroid carcinomas; F-PTCs: follicular variant of papillary thyroid carcinomas; C-PTCs: classical form of papillary thyroid carcinomas; ATCs: anaplastic thyroid carcinomas; NATs: normal adjacent tissues.

To investigate the involvement of CHD4 in *BRAF^V600E^* tumors, we analyzed the expression of CHD4 protein by immunohistochemistry staining in malignant thyroid tissues from patients and their paired NATs (see Figure 1a). Our findings revealed that 50% (14/28) of the C-PTCs, 38.5% (5/13) of F-PTCs, and 33.3% (1/3) of ATCs overexpress CHD4 protein (H-score ≥ 60) compared to their NATs, where we note a weak expression of CHD4 protein (H-score < 60) (*p*-value < 0.0001) (see Figure 1b). In the aim to correlate CHD4 protein expression with *BRAF^V600E^* status in this cohort, we explored the recently published data of our group with this same cohort [10]. Our results showed that 70% (7/10) of thyroid tumor tissues carrying the *BRAF^V600E^* mutation present a high level of CHD4 protein expression, confirming the positive correlation between CHD4 and *BRAF^V600E^* mutation in C-PTCs (see Figure 1c).

### CHD4 mRNA expression in thyroid tumor subtypes (TCGA cohort)

**Figure 2:**
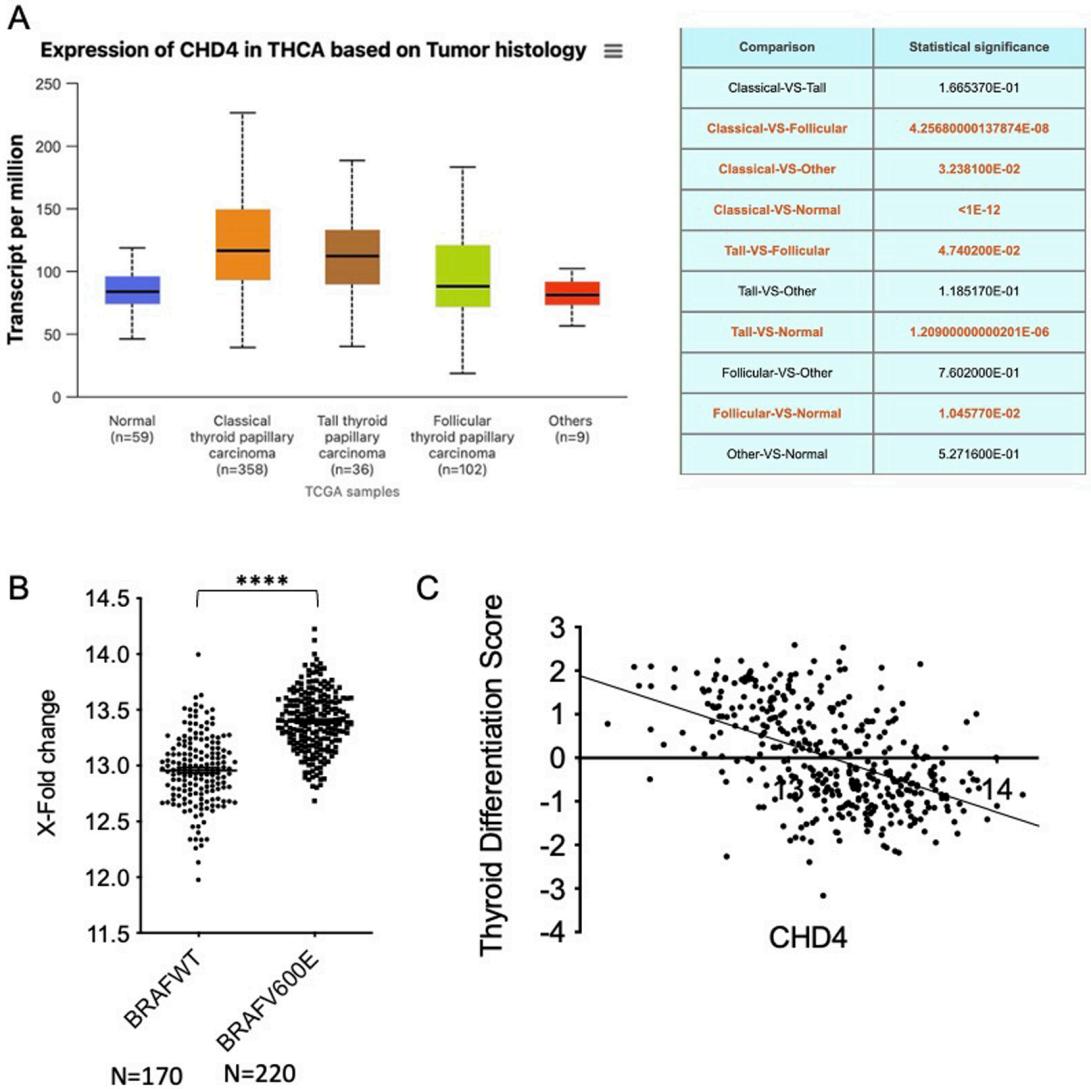
**High level of CHD4 RNA in PTC harboring *BRAF^V600E^* mutation, which is correlated with a low thyroid differentiation score.** **A.** UALCAN portal analysis of thyroid cancer samples based on the TCGA database. Expression of CHD4 for different tumor histologies of TC samples. Statistical values (*t*-test) from comparison with the normal group are indicated. **B.** Correlative analysis between CHD4 mRNA level and *BRAF^V600E^* mutation in 390 PTCs (*t*-test, *p* < 0.0001). **C.** Correlative analysis between CHD4 mRNA level and thyroid differentiation score in 390 PTCs (Pearson correlation *r =* –0.497*8*, *p* < 0.0001). CHD4: chromodomain-helicase-DNA-binding protein 4; PTCs: papillary thyroid carcinomas.

In the aim to validate our results obtained on a protein level from human thyroid tissue samples, we analyzed CHD4 mRNA expression through in several histological subtypes of thyroid cancer using the TCGA cohort (UALCAN portal analysis of thyroid cancer: 358 classical thyroid papillary carcinomas; 36 tall thyroid papillary carcinomas; 102 follicular thyroid papillary carcinomas; 9 others and 59 normal thyroid tissues). In this context, we observed a significant increase in CHD4 expression in PTC compared with normal thyroid tissues (see Figure 2A). Among the PTC subtypes, the classical and tall cell variants exhibit the highest levels of CHD4 expression, with the tall cell variant displaying the highest median expression, which corresponds to its more aggressive phenotype (see Figure 2A). In contrast, follicular thyroid papillary carcinoma exhibits significantly lower CHD4 expression than tall cell PTC, with a moderate increase compared to normal tissues (see Figure 2A). Comparisons between thyroid tumor subtypes show significant differences between follicular and tall cell PTC, although no statistically significant difference is observed between classical and tall cell PTC subtypes (see Figure 2A). Furthermore, analysis of CHD4 mRNA expression in comparison with the BRAF profile shows that CHD4 is overexpressed in thyroid tumors harboring the *BRAF^V600E^* mutation compared to thyroid tumors *BRAFwt* (see Figure 2B). This CHD4 overexpression is thus correlated with a low thyroid differentiation score (see Figure 2C).

### Association between levels of CHD4 protein expression and clinicopathological parameters of thyroid carcinomas

The comparative analysis between the levels of CHD4 protein expression and clinicopathological characteristics of 44 thyroid carcinomas (28 C-PTCs, 13 F-PTCs, and three ATCs) (Table 2) demonstrates that the over-expression of CHD4 was found to be associated with an age < 45 (55.6%: 10/18 show an H-score ≥ 60) and tumor size ≤ 1cm (83.3%: 5/6 show an H-score ≥ 60). The level of CHD4 protein expression was significantly higher within tumors with aggressive features of thyroid cancer, such as the presence of vascular emboli (57.1%: 4/7 show an H-score ≥ 60) and capsular breach (54.5%: 6/11 show an H-score ≥ 60), suggesting a role of CHD4 in thyroid tumor progression. However, a low level of CHD4 protein expression was positively correlated with lymph node metastasis (66.7%: 2/3 show an H-score < 60). Oppositely, Gender did not appear to be influenced by levels of CHD4 protein expression (Table 2).

**Table 2:** CHD4 protein expression and clinicopathological parameters of thyroid carcinoma.

**Clinico-pathological parameters**		***n* (%)**	**CHD4 overexpression (H-score: ≥ 60)**	**CHD4 low expression (H-score: < 60)**	**Chi-square/Fisher’s exact test (*p*-value < 0.05)**
**Gender**	Female	37/44 (84.1%)	17/37 (45.9%)	20/37 (54.1%)	0.7761
	Male	7/44 (15.9%)	3/7 (42.9%)	4/7 (57.1%)	
**Age**	< 45	18/44 (40.9%)	10/18 (55.6%)	8/18 (44.4%)	< 0.0001
	≥ 45	17/44 (38.6%)	8/17 (47.1%)	9/17 (52.9%)	
	unknown	9/44 (20.5%)	2/9 (22.2%)	7/9 (77.8%)	
**Histological variant**	C-PTCs	28/44 (63.6%)	14/28 (50%)	14/28 (50%)	< 0.0001
	F-PTCs	13/44 (29.6%)	5/13 (38.5%)	8/13 (61.5%)	
	ATCs	3/44 (6.8%)	1/3 (33.3%)	2/3 (66.7%)	
**Tumor size**	≤ 1 cm	6/44 (13.7%)	5/6 (83.3%)	1/6 (16.7%)	< 0.0001
	> 1 cm	27/44 (61.3%)	10/27 (37%)	17/27 (63%)	
	unknown	11/44 (25%)	5/11 (45.5%)	6/11 (54.5%)	
**Vascular emboli**	Presence	7/44 (15.9%)	4/7 (57.1%)	3/7 (42.9%)	0.0657
	Absence	37/44 (84.1%)	16/37 (43.2%)	21/37 (56.8%)	
**Capsular breach**	Presence	11/44 (25%)	6/11 (54.5%)	5/11 (45.5%)	0.0893
	Absence	33/44 (75%)	14/33 (42.4%)	19/33 (57.6%)	
**Lymph node metastasis**	Presence	3/44 (6.8%)	1/3 (33.3%)	2/3 (66.7%)	0.0072
	Absence	30/44 (68.2%)	13/30 (43.3%)	17/30 (56.7%)	
	unknown	11/44 (25%)	6/11 (54.5%)	5/11 (45.5%)	

CHD4: chromodomain-helicase-DNA-binding protein 4; F-PTCs: follicular variant of papillary thyroid carcinomas; C-PTCs: classical form of papillary thyroid carcinomas; ATCs: anaplastic thyroid carcinomas.

### CHD4 and NOX4 protein expression in human thyroid tumor tissues

**Figure 3:**
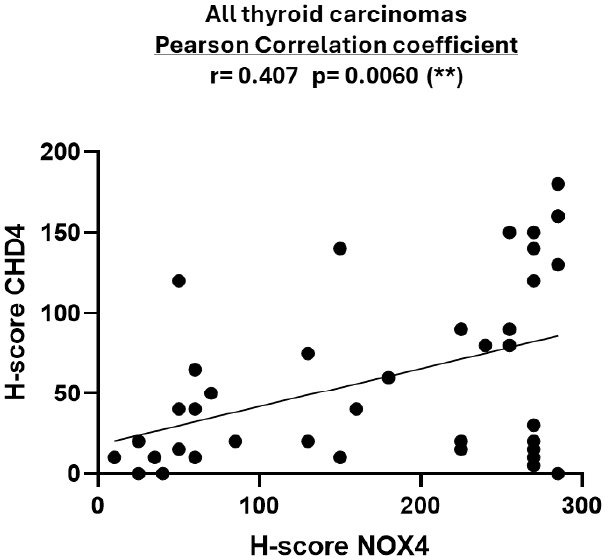
**NOX4 and CHD4 protein expression in thyroid carcinomas.** Comparative analysis between the H-scores of CHD4 and NOX4 proteins in human thyroid carcinomas [*n* = 44 (28 C-PTCs, 13 F-PTCs, 3 ATCs)]. The H-score was calculated by multiplying the score of staining intensity (0 = negative, 1 = weak, 2 = moderate, 3 = strong) by the percentage of stained cells. The highest score achieved is 300. The correlation was determined using the Pearson correlation coefficient (*r*). The statistical significance is confirmed by a *p*-value < 0.05. CHD4: chromodomain-helicase-DNA-binding protein 4; NOX4: NADPH oxidase 4.

In the aim to correlate CHD4 protein expression with NOX4 protein expression in this cohort, we explored the recently published data of our group with this same cohort [10]. CHD4 and NOX4 protein expression was assessed using the H-score. To our knowledge, our results show for the first time a statistically significant positive correlation between CHD4 and NOX4 protein expression in thyroid tumors (see Figure 3).

## Discussion

Thyroid cancer represents the most common neoplasm of endocrine gland cancers. Among thyroid cancers, PTCs are the most common type of malignancy, accounting for approximately 80% of all thyroid cancers and predominating in women, where the incidence rate is 3 times higher compared to men [1, 2, 23–25]. Histologically, PTCs include several histological subtypes that share specific nuclear features of PTCs. PTCs are mainly found in the C-PTCs and the F-PTCs, with a predominance of the classical form present in more than 50% of cases [26–28]. From a molecular point of view, each PTC variant is characterized by specific genetic alterations, and this diversity constitutes a real challenge for the management of these tumors. *BRAF^V600E^* mutation is the most common alteration in PTCs, accounting for 28 to 90% of cases [1, 3, 25, 28]. This mutation corresponds to a substitution of valine by glutamic acid at amino acid residue 600 (V600E). Consequently, this substitution results in a constitutive activation of the mutated BRAF oncogene, leading to a constitutive stimulation of the MAPK signaling pathway [1, 3–5]. The *BRAF^V600E^* mutation is mainly identified in the C-PTCs and is less commonly identified in the F-PTCs and ATC. From a clinical point of view, this mutation is associated with aggressive clinicopathological characteristics, including an advanced clinical stage, a significant risk of recurrence and mortality, and extrathyroidal and lymph node invasion in thyroid cancer [2, 29]. Epigenetic dysregulation has been observed in PTCs driven by *BRAF^V600E^*, including hypermethylation of the promoters of certain iodide-metabolizing genes such as SLC5A5 encoding for the sodium iodide symporter “NIS” [6–9, 30]; and CHD4, an epigenetic regulator of gene expression, seems to play an interesting role in thyroid cancer.

In this retrospective study, we analyzed at the protein level the expression of CHD4 in 44 malignant thyroid tissues [41 PTCs (28 C-PTCs, 13 F-PTCs), and 3 ATCs] and 42 of their NATs to determine the involvement of CHD4 in thyroid malignancy. Our results showed a highly significant level of CHD4 protein expression in malignant thyroid tissues compared to their NATs (50% of C-PTCs, 38.5% of F-PTCs, 33.3% of ATCs, and 0% of NATs) (see Figure 1b). This finding is consistent with similar results from another study carried out on a large cohort of Middle Eastern PTC patients, which revealed an overexpression of CHD4 in 46.7% of C-PTCs and 34.4% of F-PTCs [19]. Additionally, the comparative analysis between CHD4 protein expression and the presence of the *BRAF^V600E^* mutation showed a high expression of CHD4 in 70% of thyroid tumors driven by *BRAF^V600E^* (see Figure 1c). This result agrees with another study, which also showed an overexpression of CHD4 in more than 50% of PTCs-*BRAF^V600E^* [19], suggesting a positive correlation between CHD4 and *BRAF^V600E^* oncogene in thyroid cancers.

Moreover, transcriptomic analysis of the TCGA thyroid cancer cohort reflects our immunohistochemical results in human thyroid tissues, demonstrating significant overexpression of CHD4 mRNA in PTCs compared with normal thyroid tissues. Impressively, CHD4 overexpression was mostly observed in the classical and tall cell variants, with the latter displaying the highest median levels, which aligns with its well-recognized aggressive clinical behavior. The association between CHD4 mRNA overexpression and *BRAF^V600E^* mutation strengthens our protein-based observations and supports the hypothesis of a functional link between CHD4 dysregulation and *BRAFV^600E^*-driven tumorigenesis. In addition, the negative correlation between CHD4 mRNA expression and thyroid differentiation score suggests that CHD4 may contribute to tumor dedifferentiation, a key event in thyroid cancer progression and therapeutic resistance. On the whole, these transcriptomic data supply independent validation of our experimental results and highlight CHD4 as a potential biomarker associated with tumor aggressiveness and molecular subtype stratification in PTC.

In thyroid cancer, CHD4 is involved in diverse aspects of tumorigenesis, including cell proliferation, EMT, and migration [19]. The whole exome sequencing performed on 14 paired primary and metastatic PTC biopsies identified likely pathogenic mutations in genes related to DNA methylation and transcriptional repression. These mutations appear to be potentially restricted to metastatic papillary cancers, including the pathogenic CHD4 mutation, thus reinforcing the possibility of CHD4 involvement in PTCs aggressiveness [31]. Interestingly, recent data demonstrate that CHD4 upregulates *TERT* gene through its binding to the hypermethylated TET promoter in thyroid cancer cells [32]. Additionally, a bioinformatics analysis performed by Oskouie and his colleagues [33] identified CHD4 as a potential biomarker for the prognosis of PTCs. Furthermore, in the U2OS cell line, CHD4 was shown to be recruited to DNA damage sites, leading to recruitment of DNA Methyltransferases, DNMTs, to chromatin in response to oxidative damage [20, 34]. Azouzi et al. [8] showed that ROS derived from NOX4 are involved in cell dedifferentiation in *BRAF^V600E^*-driven PTCs. Recently, we have shown, for the first time, to our knowledge, a positive correlation between NOX4 protein and *BRAF^V600E^* mutation in human thyroid tumors [10]. In this study, we performed a supplementary immunostaining of the same cohort [10] with the CHD4 antibody. In this context, we observed for the first time a statistically significant positive correlation between CHD4 and NOX4 protein expression in tumoral thyroid tissues (see Figure 3), suggesting that both CHD4 and NOX4 could be positively regulated by a shared signaling pathway in thyroid carcinomas. Taken together, the findings of our study suggest a newly identified link between CHD4 and NOX4, which may serve as a potential mechanistic link between epigenetic regulation and oxidative stress in PTCs. Additionally, the relationship of CHD4 with NOX4 offers new insights into how *BRAF^V600E^*-driven signaling might coordinate chromatin remodeling and redox imbalance, thereby promoting tumor progression. Taking into consideration the established role of NOX4-derived ROS in tumor dedifferentiation and aggressiveness [8], CHD4 may contribute to the pathogenesis of PTC not only through transcriptional repression and DNA methylation but also via redox-related signaling pathways. Interestingly, the TCGA data analysis showed a negative correlation between CHD4 expression and thyroid dedifferentiation (Figure 2C). On the other hand, based on a clinical and diagnostic point of view, the analysis of CHD4 overexpression in association with NOX4 and *BRAF^V600E^* status could provide a complementary biomarker that enhances molecular stratification and improves the characterization of tumor behavior. However, the clinical utility of this approach needs to be validated by other studies.

In conclusion, these results suggest a potential involvement of these two actors in thyroid cancer development and could open a new perspective in the management of PTCs. NOX4 and CHD4 could be involved in epigenetic instability associated with thyroid cancers. The molecular mechanisms interconnecting NOX4 and CHD4 in appropriate cell line models of PTCs deserve to be illustrated.

## Abbreviations

CHD4: chromodomain-helicase-DNA-binding protein 4
C-PTCs: classical form of papillary thyroid carcinomas
ddPCR: digital droplet PCR
EMT: epithelial-mesenchymal transition
F-PTCs: follicular variants of papillary thyroid carcinomas
NATs: normal adjacent tissues
NIS: natrium iodide symporter
NOX4: NADPH oxidase 4
NuRD: nucleosome remodeling and deacetylation
PTCs: papillary thyroid carcinomas
ROS: reactive oxygen species
SLC5A5: solute carrier family 5 member 5
U2OS: human bone osteosarcoma epithelial cells

## Declarations

### Availability of data and materials

Data sharing is not applicable due to ethical restrictions and due to the possible future exploration of this cohort.

### Copyright

© The Author(s) 2026.
